# Modern Epidemiology, 4th edition. TL Lash, TJ VanderWeele, S Haneuse, KJ Rothman. Wolters Kluwer, 2021

**DOI:** 10.1007/s10654-021-00778-w

**Published:** 2021-07-03

**Authors:** Anders Ahlbom

**Affiliations:** Department of Epidemiology, Institute of Environmental Medicine, Karolinska Institutet, Solna, Sweden

Epidemiology is defined by its subject matter rather than by its methods, it says in the book, just like other branches of science. Yet, a textbook in epidemiology is typically about methods and theory which may have spurred confusion. The first sentence in the book states that epidemiology is the science that studies disease occurrence and health states in human populations. Descriptive epidemiology measures how disease frequency and other health indicators vary with time, place, and person; MacMahon, who used a similar definition of epidemiology in his groundbreaking textbook, organized parts of it according to these parameters. Etiologic, or analytic, epidemiology assesses the effect of exposures, including causes, on disease frequency. Although the vast majority of academic epidemiology seems to belong to the second category, Modern Epidemiology emphasizes the need for descriptive studies. However, it also states that there is often no clear border between descriptive and analytic epidemiology, and they are not treated separately in the book, except that there is a chapter about surveillance.

Modern epidemiology started to develop only in the middle of the twentieth century, the book says, although some examples of sophisticated epidemiology had appeared earlier. It is a reasonable guess that the development of modern epidemiology started because new research questions required new tools. This was when epidemiology started to investigate diseases that manifested long time after start of exposure, where the outcome was rare, and multiple causes were abundant. Epidemiology gradually turned into a scientific discipline in its own rights.

Fundamental concepts needed to be defined and also named such that they could be used in reports and discussions, and it was a sine qua non for the turn of epidemiology into a distinctive discipline. Given how epidemiology is defined the most fundamental concepts are those that are used to measure disease frequency. It is a little disappointing to note that there still is not a unified terminology in this respect and that the authors sense a need to offer alternative terms for core concepts. It is a relief to see that the awkward cumulative incidence is gone and replaced with the logical incidence proportion, but prevalence is still prevalence and not, the analog, prevalence proportion. It is noteworthy that the book spends 25 pages on measures of incidence and prevalence, a topic that may seem rather trivial, and perhaps had been so if the measures had only occurred in life table like models. But reality makes things complicated because the populations and situations where these measures will actually be calculated vary widely and are often unfriendly to epidemiology. The involved complexities must be appreciated to get the numbers right but are often downplayed in books and teaching.

Right after the introductory chapter that defines epidemiology and before everything else are two chapters about causal inference, one about reasoning embodied in canonical frameworks, as the authors phrase it, and one about mathematical models of causation, including directed graphs and also the classical pie models. The prominent placement of these topics is logical but also a testament to the central and explicit role that causality has been given in epidemiology. These chapters are succinct but contain a wealth of ideas about causation and its assessment. Interestingly enough, though, the authors hold back from defining causation or causal variable. Indeed, the book says that what constitutes a cause remains an issue of debate and discussion. One of the topics is whether a variable must be manipulable to be considered causal. This is a rather novel issue in epidemiology and stems from the integration of modern causal inference. No answer is given in the book.

The parts about designs of study reflect the development of modern epidemiology that has taken place since the middle of the previous century. These parts include the ideas behind the cohort study, but perhaps more typically the ideas about the case–control study ending with the case–control study being almost a special case of the cohort study. And the case-crossover study being a special case of the case–control study. This is trivial to the readers of Modern Epidemiology, but a recent book by a prominent science writer explained that the case–control study is an extension of the case study. The study design parts of Modern Epidemiology include a large number of other aspects such as data acquisition, measurement and selection bias, including principles for quantification of the resulting bias. This goes beyond the scope of study design in most other textbooks.

A big part of the book is directed to data analysis and statistical methodology. Since there is an abundance of excellent textbooks in statistics and in biostatistics the need to put this part into this book of modern epidemiology deserves a comment. There appears to be two separate explanations that drive this. First, the data, study designs, and required outputs are often such in epidemiology that special methods are needed that are typically not included in standard statistical books. Indeed, several statistical methods were developed specifically for use in epidemiologic data analysis. Examples mentioned in Modern Epidemiology are the Mantel–Haenszel methods that were presented 70 years ago and served epidemiology well for decades and the logistic regression modelling developed somewhat later and still one the most used tools in the box. Second, epidemiology is dependent on data and numbers and highly sensitive to the way that data and results are analyzed and interpreted. This has led to careful considerations of how to look at data, including how to assess random errors. These considerations are more specific and sometimes different from what standard textbooks in statistics present. One essential part is the strict advice to avoid simple testing of null-hypotheses. This is one of the points that were made already in the first edition of Modern Epidemiology, and that has been repeated ever since in various wordings. The *P*-value function is now central to this discussion. This function offers not only the common *P*-value but also the *P*-value for any given alternative hypothesis, and not only that, it also provides confidence intervals for any given level of confidence. Indeed, a remarkable function. The statistics part of the book includes a series of topics that are not easily found in other textbooks and definitely not in one place. These include Bayesian statistics, interaction analysis, mediation analysis, ecological analysis, and more.

The Introduction describes the history of Modern Epidemiology and the thinking behind the 4th edition. The first edition came out 35 years ago, was not big, and was single authored by Kenneth Rothman. The current book has four authors with Timothy Lash as first name and Rothman as last. In addition, 35 more authors have contributed to various sections of the book. The last page of my printed version is numbered 1,174, so this is not a small book. Hence, there has been quite a transition over the years. The additional 35 names have provided rather distinct, concrete, and practical insights into a number of special topics that take the core theoretical parts of the book forward and helps to bridge the gap to applications. Some of these special topics are infectious disease epidemiology, clinical epidemiology, social epidemiology, and pharmacoepidemiology. Modern Epidemiology 4th is indeed a comprehensive book. The one thing I had expected to find in the book but that wasn’t there is a discussion about meta-analysis and pooling of studies, but I don’t suppose the reason for its absence is that the authors forgot to put it in.

Modern Epidemiology 4th is a gigantic project completed in grand style. One can only imagine all the considerations and discussions about content, terminology, ordering, et cetera that the authors had to go through and agree on to make this edition happen. It is an easy prediction that this will be the standard textbook in all academic institutions for a long time to come and that some will read the entire book, but more will use it as a reference and encyclopedia. For a number of topics, what this book presents will most likely be the end of story. This applies in particular to the core chapters, that were also in all prior editions, but expanded, developed, and matured over the 35 years. Core principles behind measures, study design, and data analysis belong there. But epidemiology continues to develop and change. Currently we are in the middle of the integration and adaptation of causal modelling and causal inference. There are also areas that we only have started to see above the horizon that may call for a 5th edition, such as use of artificial intelligence and big data in epidemiology.

